# Domestic

**Published:** 1841-07-10

**Authors:** 


### DOMESTIC.

*Case of Popliteal Aneurism—Operation—Cure.* By A. W. SHIPMAN, M.D.—Some time in the month of June, 1839, I was consulted by Nicholas Dyer, of Willet, Cortland County, N.Y., for a tumor in the ham of the right leg. The patient was about 30 years of age, of a good constitution, and a farmer by occupation. The history which he gave was, that in the month of April previous, he began to feel some pain and soreness in the ham, which he attributed to rheumatism, and accordingly used some domestic application, but without any effect. In May he discovered a tumor in the ham, for which he used some volatile liniment, but the swelling continued to increase, and he applied to Dr. Lyman Eldridge, of Cincinnatus, who advised him as to the nature of his disease, and recommended him to consult me. At the time I first saw him, which was about the 10th of June, I found a tumor occupying the popliteal space, as large as an orange; it was tender on pressure, and pulsated pretty strongly, which ceased when pressure was made over the artery in the upper part of the thigh. The leg was flexed and could not be extended, the attempt giving him great pain. The tumor was hot, and the skin inflamed over it, as well as the calf of the leg. The foot was œdematus and numb. He suffered much pain day and night, and the tumor was rapidly increasing in size; it was difficult for him to walk. Under these circumstances I did not hesitate to advise him to submit to an operation as soon as possible.

This I performed on the 18th of June, with the assistance of Drs. Eldridge, McWhorter and Briggs, of Cincinnatus in the usual manner, at the upper third of the thigh. The artery was separated as little as possible from its surrounding connections, and one firm silk ligature applied. The pulsations in the tumor immediately ceased; it became reduced nearly one half in size; the leg and foot became cold and pale, with a slight increase in the numbness, which already affected the limb. Hot flannels were placed around the leg and foot, and bottles of hot water to the foot and sides of the leg. In a few hours, re-action came on, with an increase of temperature in the leg and foot. No untoward symptoms came on to interrupt the cure. The ligature separated on the 20th day from the operation, the wound healed, the tumor in the ham was absorbed entirely in the course of a month, the leg could be extended perfectly, and its strength returned in a few months. He is now, and has been, as well as before the disease made its appearance.—*Boston Med. and Surg. Jour.*

---

*Case of Aneurism at the bend of the arm from a wound—Operation—Cure.* By A. W. SHIPMAN, M.D., Pres. of Cortland Med. Soc'y.—A son of Roswell Ackley, of Groton, Tompkins Co., N. Y., æt. 15, was wounded with the point of a penknife at the bend of the arm, about the first of October, 1840. There was a jet of blood to the distance of six feet at the time, but some of the bystanders made compression over the orifice and arrested the hæmorrhage. A physician was called, who applied compresses and a bandage, and kept it tightly done up for a week, when the dressings were removed, and the boy began to make some use of his arm, when hæmorrhage again recurred from the wound (which had not healed) to some considerable amount. It was again bandaged, but it continued to bleed from time to time. The whole of the forearm was swollen and discolored from infiltration of blood, and a tumor formed over the seat of the injury, which continued slowly to increase in size. About this time a *steam doctor* saw it and promised a speedy cure by the application of stimulants and frictions, which on trial only served to increase the size of the swelling, and augment the frequency of the hæmorrhages. I was requested to visit him on the 28th of October. Found the forearm in a state of semiflexion, much swollen and discolored, and a tumor in the bend of the arm as large as the fist, with a small orifice (the original puncture unhealed) near the apex. There was no pulsation in it to be discovered, nor could I learn that there had ever been. No pulsation in the radial artery could be perceived at the wrist. The fingers were flexed and could not be easily extended.

With the assistance of Dr. Daniel Haven, I proceeded to the operation by cutting down upon the brachial artery about midway between the axilla and bend of the arm, at the edge of the biceps, and carefully separating the media nerve and disturbing as little as possible the surrounding connections; one firm silk ligature was applied; the wound closed by adhesive plaster, and the arm placed in a sling, carefully wrapped in cotton. No unpleasant symptoms followed. The ligature came away on the 14th day from the operation. A partial suppuration took place in the tumor, which with some coagula was discharged from the puncture. After this, frictions with stimulating liniments completely dispersed the remainder of the swelling. The arm and fingers became straight, and he speedily regained their strength and use.

How infinitely preferable this simple, yet efficient operation, to the method formerly pursued, and sometimes recommended in these days, of opening the aneurismal sac and searching for the wounded vessel, and tying both above and below the wound.

*Cortlandville, N. Y., June 16th,* 1841.

*Ibid.*

---

*Aleppo Button.*—The Rev. Mr. Thomson, a missionary residing in Syria, has transmitted to this country, through the Missionary Herald of the present month, an account of a singular disease well known in that country, which we do not recollect of having met with in any other publication. Mr. Thomson premises by remarking that Aleppo has the reputation of being a healthful residence. The air is cold in winter, and occasionally quite frosty. Neither lemons nor oranges succeed there, on account of the severity of the weather. Ice sometimes forms several inches in thickness. In summer the heat occasionally rises to 105 degrees of Fah. but is usually followed by a delightful evening breeze. The climate is regarded far superior in Aleppo to that in Beyroot.

A singular disease known to the foreign residents, as well as natives, greatly to be feared, is called the *Aleppo button.* It seems almost peculiar to that place; indeed, it is questionable whether it has ever been developed in any other place in all Syria. Both the nursing babe and the aged are alike the subjects of the malady. Although it may exist on any part of the body, the hands and face are commonly the seat of its development. Persons residing there soon have the button—the natives rarely ever escape, although instances occur where the individual escapes its attack until many years after strangers have returned to their distant homes in other countries. Another singular fact about the Aleppo button, is the well-established one, that just one year is required to run its course—it is six months in coming, and six more in fairly going off—and that with surprising regularity. Medical treatment seldom does any good, and it may do much injury. Another queer law regulating the Aleppo button is this, viz.: there is both a male and a female button. The male button is recognised by one scab, not very painful. On the contrary, the female button produces many sores or ulcers. By inoculation it is thought possible to produce either—the experiment having been repeatedly tried by one of the Pasha's doctors. If this should be subsequently verified, since all going into the city must sooner or later have the button, they may fix upon any locality on their own bodies which will incommode them the least. Some pretty faces have been sadly scarred by its painful inroads. Neither the cause, or a certain and speedy mode of stopping the progress are known, yet it is believed by intelligent persons that some singular chemical condition of the water has an agency in the matter. However, at Antab, says Mr. Thomson, and at Bagdad, there is recognised a similar disease, varying perhaps only in the imagination of the suffering patient.—*Ibid.*

